# Safety of dietary nitrate supplementation by calcium nitrate for finishing pigs as measured by methemoglobin and serum and tissue nitrate levels

**DOI:** 10.1093/tas/txad135

**Published:** 2023-11-30

**Authors:** Amy M Sheppard, Jennifer L G van de Ligt, Padmakumar Pillai, Christine M Crincoli, Richard J Faris, Molly L McGhee, Brent R Frederick

**Affiliations:** ToxStrategies LLC, Milwaukee, WI 53201, USA; ToxStrategies LLC, Minneapolis, MN 55111, USA; Research and Development, Cargill, Inc., Wayzata, MN 55391, USA; Research and Development, Cargill, Inc., Wayzata, MN 55391, USA; Animal Nutrition and Health, Cargill, Inc., Wayzata, MN 55391, USA; Animal Nutrition and Health, Cargill, Inc., Wayzata, MN 55391, USA; Animal Nutrition and Health, Cargill, Inc., Wayzata, MN 55391, USA

**Keywords:** dietary nitrate, methemoglobin, safety, swine, tissue levels

## Abstract

Nitrate supplementation has been studied as a beneficial constituent of the human diet, particularly for its effects on vascular health through vasodilation. Recent studies have focused on the benefits of nitrate supplementation in animals, especially in swine. Up to 1,200 mg/kg dietary nitrate supplementation from Ca nitrate was beneficial in farrowing and lactating sows and their offspring, and up to 6,000 mg/kg supplemental nitrate showed no adverse health effects in sows or piglets. Controlled study data evaluating the safety of nitrate supplementation to growing swine of any weight class is scant. Therefore, an experiment was conducted to test the hypothesis that increased inclusion rates of dietary nitrate through the addition of Ca nitrate in diets would not influence concentrations of nitrate or nitrite in serum and tissue, nor blood hemoglobin and methemoglobin. Forty-eight individually housed pigs (initial weight 119.1 ± 5.3 kg) were randomly allotted to four dietary treatments containing 0, 500, 1,000, or 2,000 mg/kg dietary nitrate and fed experimental diets for 28 d. Growth performance was not influenced (*P* > 0.10) by dietary treatment. The most sensitive safety endpoint, methemoglobin, did not change (*P* > 0.10) with dietary nitrate exposure up to 2,000 mg/kg. Serum and tissue nitrate and nitrite levels, myoglobin, and hemoglobin were not adversely affected (*P* > 0.10). Total myoglobin in the loin linearly increased (*P* < 0.05) with greater dietary nitrate in the diet, which is correlated with the red color of meat. This work established the safety of up to 2,000 mg/kg dietary nitrate from Ca nitrate as an ingredient in food for finishing pigs.

## Teaser

This work established the safety and benefit of up to 2,000 mg/kg dietary nitrate from calcium nitrate as an ingredient in feed for finishing pigs. Growth performance and methemoglobin, the most sensitive safety endpoint, were not affected whereas total myoglobin in the loin, which is correlated with the red color of meat, linearly increased with greater dietary nitrate in the diet.

## Introduction

Previous research has shown nitrate supplementation to be beneficial for vascular function by acting as a nitric oxide (**NO**) precursor (Lundberg and Govoni, 2004; Lundberg et al., 2008). Additionally, numerous reviews have summarized the benefits of dietary nitrate in humans (Bryan and Loscalzo, 2017; Wikoff et al., 2018), particularly for cardiovascular health. Historically, from a toxicology perspective, dietary nitrate has been considered more of a contaminant in the human diet because of reports of methemoglobinemia in infants exposed to well water with high nitrates in the mid-20th century (Comly, 1945; Bosch et al., 1950; Walton, 1951; USEPA, 1991) that were also contaminated with other compounds that may have contributed to the methemoglobinemia (Avery, 1999; Powlson et al., 2008; Gilchrist et al., 2010). However, work by Wikoff et al. (2018) demonstrated that the benefits of nitrate exposure outweigh the risks in humans.

Although studies have shown that dietary nitrate may be beneficial, there also have been concerns regarding nitrate toxicity in swine (Smith et al., 1959; Emerick, 1974; Bruning-Fann and Kaneene, 1993; Nyachoti and Kiarie, 2010; Doepker et al., 2021). Many of the favorable physiological effects from dietary nitrate relate to its role as an NO precursor where NO has been shown to be an endothelium-derived relaxing factor that produces vasodilation (Lundberg and Govoni, 2004) and influences angiogenesis (Gladwin and Kim-Shapiro, 2008), bone growth (Wimalawansa, 2020), and metabolic rate (Pawlak-Chaouch et al., 2016). In swine, nitrate is reduced to nitrite, which when absorbed, can combine with red blood cells and oxidize the ferrous iron in hemoglobin, thereby forming methemoglobin (MetHb). MetHb is a molecule with limited oxygen-carrying capacity, and the rate of formation depends on the rate at which nitrate is reduced to nitrite (EFSA, 2020). Methemoglobinemia is defined as a level of 10% methemoglobin in the blood of humans, as a percentage of total hemoglobin (Wright et al., 1999), and early signs of nitrate toxicosis in animals appear at 30% to 40% methemoglobin (Thompson, 2022). In 2020, EFSA evaluated existing data and identified a no-observed-adverse-effect level (**NOAEL**) of nitrate exposure of 410 mg/kg body weight/day for pigs, and in 2021, Doepker et al. identified a higher NOAEL for nitrate, between 600 and 800 mg/kg body weight/day using methemoglobin blood measurements and the manifestation of methemoglobinemia as the most sensitive endpoints.

A study evaluating the safety of nitrate supplementation via Ca nitrate in sows shortly before farrowing and throughout lactation documented no adverse effects, including the absence of methemoglobinemia, at dietary levels of up to 6,000 mg/kg nitrate (van de Ligt et al., 2021). The studies establishing safety of 6,000 mg/kg dietary nitrate in sows provided 53.9 to 70.0 mg/kg body weight/d nitrate in late gestation with sow body weight about 570 to 600 lbs and 121 to 217 mg/kg body weight/d nitrate in lactation with sow body weight about 490 to 525 lbs (van den Bosch et al., 2019a; van de Ligt et al., 2021). However, there are no controlled studies evaluating the impact of supplemental dietary nitrate on methemoglobin concentrations or serum and tissue levels of nitrate in growing swine approaching slaughter weight. The tissue levels of nitrate are of particular concern due to the public health impact of changing dietary nitrate intake for humans.

In consideration of the potential risks and benefits of dietary nitrate supplementation, the primary objective of this study was to test the hypothesis that increased inclusion rates of dietary nitrate through the addition of Ca nitrate in diets would not influence serum and tissue nitrate and nitrite levels, hemoglobin, methemoglobin, and myoglobin forms in finishing pigs.

## Materials and Methods

The study was conducted at the Cargill Animal Nutrition Global Innovation Center in Elk River, MN, USA. The pigs were managed in accordance with the guidelines described in the *Guide for the Care and Use of Agricultural Animals in Research and Teaching*, 4th edition (2020). The study protocol was approved by Cargill’s Institutional Animal Care and Use Committee.

### Experimental Diets

Four experimental diets were formulated to meet or exceed the nutritional requirements of finishing pigs as suggested by the Cargill Nutrition System; these nutritional requirements also meet or exceed the National Research Council’s estimated requirements for pigs (NRC, 2012). Four complete diets based primarily on corn, wheat middlings, and soybean meal were manufactured to contain increasing concentrations of Ca nitrate (CAS 10124-37-5, ADOB, Poznan, Poland) at the expense of corn: 0%, 0.08%, 0.16%, or 0.32% Ca nitrate, which provided 0, 500, 1,000, or 2,000 mg/kg supplemental dietary nitrate, respectively. These supplemental nitrate levels are equivalent to 0, 4.2, 8.4, or 16.8 mg/kg body weight, respectively, based on the average initial weight of the swine. Diets were fed in pelleted form (4 mm). Local well water was provided to the pigs, and water nitrate (<2.2 mg/kg nitrate) was measured by Cumberland Valley Analytical Services, Inc. (Waynesboro, PA, USA) by 4500-NO3-D. Nitrate Electrode Method (Standard Methods Committee, 2019).

### Animals, Housing, and Experimental Design

Forty-eight pigs (119.1 ± 5.3 kg; 24 gilts and 24 barrows) that were the offspring of Camborough sows and Line 327 boars (Pig Improvement Company, Hendersonville, TN, USA) were stratified by body weight and sex and randomly allotted to one of four dietary treatments in a randomized complete block design. Pigs were fed treatment diets for 28 d in April and May 2022. The mixed-sex pigs were identified by ear tag and were ~18 weeks post-weaning at study initiation. Pigs were housed in two environmentally controlled rooms in individual pens (1.07-m × 1.98-m pens). Each pen contained a self-feeder that allowed ad libitum intake and a nipple waterer. The pens had slatted, concrete flooring and no enrichment devices. The room temperature was controlled at 16.7 to 21.1 °C, and the lighting program provided a 12:12 (L:D) h cycle. Humidity was not controlled; however, a 22.9-cm fan was operated in each room to provide a minimum airflow of 0.3 m^3^/min, with the fan’s speed increasing as the temperature increased to regulate the environment. The room temperature was monitored twice daily, at which time the pigs were monitored for overall wellness and any apparent clinical signs of potential nitrate toxicity. Specifically, the animals were observed for difficulty in breathing, incoordination, blue to brown mucous membranes, and/or abnormal urination. In the event of an abnormality, monitoring frequency increased, and a veterinarian was notified if needed.

Pig body weight was measured on day 0 and day 28 to calculate body weight gain. Feed intake was calculated as the total amount of the feed provided minus the amount of the feed remaining in the feeder on day 28. Feed efficiency (**G:F**) was calculated as average daily gain (**ADG**) divided by average daily feed intake (**ADFI**).

### Sample Collection and Analyses

Feed samples were analyzed for nitrate using a method based on EPA Method 353.2 (automated cadmium reduction) by Midwest Laboratories (Omaha, NE, USA). Under this method, the nitrate was reduced to nitrite, and reported as nitrate/nitrite N, with a detection limit of 20 mg/kg. Moisture and crude protein in the complete diets were estimated by near-infrared spectroscopy using calibrations developed from wet chemistry for moisture (method 903.15; AOAC Int., 2007) and crude protein (method 990.03; AOAC Int., 2007).

After being weighed on day 28, the pigs were fasted for 14 to 16 h overnight. On day 29 prior to euthanasia, blood was collected via jugular venipuncture. Two blood samples were collected in a vacutainer containing the anti-coagulant ethylenediaminetetraacetic acid (**EDTA**), and a third blood sample was collected in a serum separator Vacutainer. Whole blood samples were stored indirectly on ice to keep chilled but not frozen between the time of collection and analysis. Hemoglobin and methemoglobin were analyzed from whole blood samples collected with EDTA within 36 h of collection. Hemoglobin was analyzed with a SYSMEX XT-V automated hematology analyzer by IDEXX (Sacramento, CA, USA), and methemoglobin was analyzed by the Iowa State University Veterinary Diagnostic Lab (Ames, IA). Serum was collected from centrifuged tubes within 1 hour of sample collection and stored in a −80 °C freezer until analysis. Serum nitrate and nitrite were analyzed in serum samples by IDEXX (Sacramento). The analytical limit of quantification for nitrate was 10 mg/kg, and the limit of detection was 1 mg/kg. Nitrite is not tested routinely, and measurements were reported as none detected, positive, or strongly positive.

Following blood collection on day 29, animals were euthanized by penetrating captive bolt and exsanguination. Liver and loin (longissimus dorsi) samples were collected, diced into 1 cm cubes, snap-frozen in liquid nitrogen, and stored at −80 °C until analysis. Liver and loin samples were analyzed for nitrate and nitrite content by Midwest Laboratories (Omaha). Nitrate analysis for the tissues was based on EPA Method 353.2, whereby the nitrate was reduced to nitrite and reported as nitrate/nitrite N, with a detection limit of 20 mg/kg. Nitrite was analyzed based on method 973.31 (AOAC Int., 2007), with a detection limit of 5 mg/kg, and was reported as sodium nitrite. The nitrate level was then estimated by converting the measured sodium nitrite to sodium nitrite N and subtracting this value from nitrate/nitrite N.

Depending on the oxidative state, myoglobin, like hemoglobin, is present in the redox forms of deoxymyoglobin, oxymyoglobin, and metmyoglobin. All forms were measured to determine whether the supplemental dietary nitrate influenced the level of myoglobin or the ratio of the various redox forms of myoglobin in the loin. Myoglobin and myoglobin redox forms were measured in loin samples using methods from the American Meat Science Association (2012) and Tang et al. (2004). To prepare the samples, 5 g of loin was homogenized in 25 mL of ice-cold 40-mM potassium phosphate buffer (pH 6.8) and stored on ice. After one hour, the frozen loin samples were centrifuged for 30 minutes at 17,000 × *g* and filtered through a 0.4-µm syringe filter, from which the supernatant was collected. A SpectraMax M2e (Molecular Devices, San Jose, CA, USA) was used to measure absorbance at 503, 525, 557, 582, and 700 nm. The validity of both the assay and the chosen absorbances was measured using pure myoglobin (M0630, Sigma-Aldrich, St. Louis, MO, USA) alone, or spiked in meat samples, to determine the accuracy in quantifying the myoglobin. The absorbance at 525 nm was used to calculate the total myoglobin content of the loin samples. The absorbances of 503, 557, and 582 nm were used to calculate metmyoglobin, deoxymyoglobin, and oxymyoglobin, respectively, as a percentage of total myoglobin. The absorbance of 700 nm was used as a background correction for turbidity. The validity of the assay used to measure the myoglobin content of the meat samples was tested first by measuring pure myoglobin alone, then by measuring myoglobin spiked in meat samples. The results of running these control samples indicated a method recovery between 78% and 88%, which is within the range published previously by Liu et al. (2021).

### Statistical Analysis

Data were analyzed in the software R (version 4.1.1; Vienna, Austria). Pen was considered the experimental unit (*n* = 12 per treatment). Unless specifically noted, data were analyzed as a general linear model with treatment as the fixed effect and weight block and sex as random effects, and treatment least square means were reported. Initial body weight was used as a covariate for growth performance data. Statistical relevance was defined as *P < *0.05. Linear and quadratic contrasts were constructed to determine the effect of nitrate inclusion rate on variables of interest.

## Results

The analyzed composition of the feed, including the concentration of nitrate, was in line with the expected values (Table 1). No clinical signs of nitrite toxicity, such as difficulty breathing, loss of coordination, blue to brown mucous membranes, and/or abnormal urination, were noted in any pigs during the study period. No pigs were removed from the study, and no pigs died due to dietary treatment. One pig from the control treatment group (0% nitrate) was observed not consuming feed on day 19 of the study. The pig was found to have an elevated body temperature, received veterinary treatment, and resumed adequate feed intake by the following day. This issue was determined to be unrelated to the dietary treatment in the study.

**Table 1.** Composition of experimental diets containing 0, 500, 1,000, or 2,000 mg/kg dietary nitrate supplied by Ca nitrate

Item	0 mg/kg nitrate	500 mg/kg nitrate	1,000 mg/kg nitrate	2,000 mg/kg nitrate
Ingredient composition, %				
Corn	74.54	74.46	74.38	74.22
Soybean meal	6.00	6.00	6.00	6.00
Wheat middlings	16.88	16.88	16.88	16.88
Poultry Fat	1.00	1.00	1.00	1.00
Limestone	0.67	0.67	0.67	0.67
Sodium chloride	0.43	0.43	0.43	0.43
l-Lysine HCl	0.30	0.30	0.30	0.30
l-Threonine	0.07	0.07	0.07	0.07
l-Tryptophan	0.02	0.02	0.02	0.02
Vitamin–mineral premix^1^	0.10	0.10	0.10	0.10
Ca nitrate	0.00	0.08	0.16	0.32
Calculated composition				
Crude protein, %	12.00	12.09	12.18	12.28
Net energy, kcal/kg	2,500	2,497	2,495	2,492
Lys, %	0.73	0.73	0.73	0.73
SID^2^ Lys, %	0.64	0.64	0.64	0.64
Ca, %	0.30	0.32	0.33	0.35
P, %	0.37	0.37	0.37	0.37
Analyzed composition				
Crude Protein, %^3^	13.32	13.18	13.27	13.20
Moisture, %^3^	13.92	13.91	13.96	13.85
Nitrate N, mg/kg	ND^4^	110	241	487
Nitrate, mg/kg^5^	—	487	1,067	2,157

^1^Provided per kilogram of diet: vitamin A, 5,000 IU; vitamin D3, 800 IU; vitamin E, 50 IU; vitamin B12, 17.5 µg; menadione, 1.75 mg; folic acid, 0.55 mg; niacin, 25 mg; pantothenic acid, 30 mg; pyroxidine, 1 mg; riboflavin, 4 mg; thiamin, 0.75 mg; copper, 12.6 mg; iron, 77 mg; iodine, 0.49 mg; manganese, 35 mg; zinc, 77 mg; selenium 0.21 mg; phytase, 391 FTU.

^2^Standardized ileal digestible.

^3^Estimated by near-infrared spectroscopy.

^4^Not detected.

^5^Calculated from nitrate N.

Although the present experiment was not designed to detect differences in growth performance, growth performance was measured to broadly assess the overall well-being of the pigs. No significant differences in ADG, ADFI, or G:F were observed (*P* > 0.10, Table 2) with all supplemented pigs having ADG and G:F from 104% to 120% of the control pigs.

**Table 2.** Growth performance [body weights, average daily gain (ADG), average daily feed intake (ADFI), and feed efficiency (G:F)] and of finishing pigs fed 0, 500, 1,000, or 2,000 mg/kg nitrate for 28 d

	Dietary nitrate, mg/kg					*P* value		
	0	500	1,000	2,000	SEM	Model	Linear	Quadratic
Body weight, day 0, kg	119.0	118.8	119.0	119.5	4.51	0.934	0.570	0.759
Body weight, day 28, kg	141.6	146.0	142.8	143.7	4.77	0.568	0.821	0.566
ADG, days 0 to 28, kg	0.81	0.97	0.85	0.86	0.114	0.505	0.991	0.466
ADFI, days 0 to 28, kg	3.05	3.45	3.08	3.08	0.202	0.193	0.631	0.358
G:F, days 0 to 28, kg/kg	0.24	0.28	0.27	0.27	0.035	0.659	0.680	0.317

The primary objective of the trial was to assess the safety of nitrate from Ca nitrate in pigs, which was accomplished by measuring hemoglobin and methemoglobin in blood as well as nitrate and nitrite in serum, loin, and liver (Table 3). No significant differences were detected for blood hemoglobin and methemoglobin (*P* > 0.10). Serum, liver, and loin nitrate and nitrite analyses are reported as number of samples within each group of 12 samples with analyzed values above the detection limit. Serum nitrite levels were undetected in the samples for all four treatment groups. Three of the 12 serum samples collected from pigs fed 2,000 mg/kg dietary nitrate group had nitrate values above the 1-mg/kg detection limit; however, none of the serum nitrate values exceeded the quantification limit of 10 mg/kg. All liver and loin samples contained undetectable levels of nitrate or nitrite. Total myoglobin in loin samples linearly increased (*P* <* *0.05) with increasing dietary nitrate. The redox forms of myoglobin, including metmyoglobin, did not differ across dietary nitrate levels (*P* > 0.10).

**Table 3.** Effect of diet on serum hemoglobin and methemoglobin, nitrate and nitrite in serum, loin, and liver, and total myoglobin and proportion of different redox forms of myoglobin

	Dietary nitrate, mg/kg					*P* value		
	0	500	1,000	2,000	SEM	Model	Linear	Quadratic
Serum hemoglobin, g/dL	13.18	12.61	12.15	12.77	0.821	0.846	0.754	0.409
Serum methemoglobin, %	11.24	11.77	12.09	11.60	1.901	0.983	0.889	0.706
Number of samples with detectable nitrate^1^								
Serum	0/12	0/12	0/12	3/12				
Liver	0/12	0/12	0/12	0/12				
Loin	0/12	0/12	0/12	0/12				
Number of samples with detectable nitrite^1^								
Serum	0/12	0/12	0/12	0/12				
Liver	0/12	0/12	0/12	0/12				
Loin	0/12	0/12	0/12	0/12				
Total myoglobin in loin, mg/g	1.24	1.18	1.25	1.42	0.070	0.018	0.007	0.106
Deoxymyoglobin, %^2^	8	10	9	7	1.2	0.358	0.354	0.235
Oxymyoglobin, %^2^	78	71	77	78	3.7	0.515	0.731	0.414
Metmyoglobin, %^2^	11	16	12	13	2.6	0.530	0.938	0.521

^1^Data presented as the number of samples above detection limit out of total number of samples analyzed. All serum samples were below the limit of quantification for nitrate (10 mg/kg).

^2^Data presented as the proportion of total myoglobin.

## Discussion

Dietary nitrate is achieving recognition as a beneficial nutrient because of its favorable physiological effects in both humans and swine (Hord et al., 2009; van den Bosch et al., 2019a; b). Many of the favorable physiological effects from dietary nitrate relate to its role as an NO precursor with a key, but not limited, physiological role of NO as an endothelium-derived relaxing factor that produces vasodilation (Lundberg and Govoni, 2004). It has also been shown that NO influences angiogenesis (Gladwin and Kim-Shapiro, 2008), bone growth (Wimalawansa, 2020), and metabolic rate (Pawlak-Chaouch et al., 2016).

In human health, dietary nitrate supplementation has been recognized to be beneficial for vascular function and cardiovascular health (Lundberg and Govoni, 2004; Lundberg et al., 2008; Bryan and Loscalzo, 2017; Wikoff et al., 2018). Historically, from a toxicology perspective, dietary nitrate has been considered more of a contaminant in the human diet; however, the work by Wikoff et al. (2018) demonstrated that the benefits of nitrate exposure outweigh the risks in humans. Increased vascularization provided by nitrate converting to NO is beneficial to the cardiovascular system and can also provide benefits to the skeletal system. NO is synthesized naturally through the l-Arginine (Arg)-NO pathway via NO synthase (**NOS**) enzymes (van’t Hof and Ralston, 2001) and may also be generated outside the l-Arg pathway. Ghasemi (2022) concluded that even with adequate l-Arg supply, the NOS-dependent pathway is insufficient to meet the needs for all NO functions; thus, the nitrate–nitrite–NO pathway is essential. In the nitrate–nitrite–NO pathway, nitrate consumed in the diet or from enterosalivary circulation converts to nitrite through the action of commensal bacteria in deep crypts on the posterior tongue (Weitzberg et al., 2010). This nitrite is converted to NO in the stomach (Ghasemi, 2022) or absorbed into systemic circulation for bioactivation to NO in many tissues, especially under acidic and hypoxic conditions (Weitzberg et al., 2010). The systemically circulating nitrite-to-NO conversion occurs when heme proteins enter their “deoxy” state, resulting in deoxy-hemoglobin being a major source of NO signaling from nitrite (Amdahl et al., 2020). As a result, nitrite transport in the blood provides an endocrine form of NO to the periphery, where nitrate is reduced to NO during rapid hemoglobin deoxygenation at the arterio-venous interface (Gladwin and Kim-Shapiro, 2008).

Although the mechanisms for NO circulation are most researched in humans, the essentiality of NO for normal physiological processes and the shared NO metabolic pathways across mammalian species results in an understanding of the increased importance of the nitrate-nitrite-NO pathway for species where Arg may already be a limiting dietary amino acid. For example, in modern swine production, the emphasis on sustainability and environmental impact has resulted in both the use of byproducts that are low in Arg and much lower protein diets than fed in the past to reduce nitrogenous waste. When these dietary pressures are coupled with the fast growth rate of modern swine genetics, Arg, a conditionally essential amino acid (NRC, 2012), is a limiting amino acid requiring supplementation to meet a minimum requirement for growth. However, this level of dietary Arg may not be adequate to meet the more recently understood physiological needs for NO solely through the l-Arg–NO pathway (Wu et al., 2018) indicating NO from dietary nitrate through the nitrate–nitrite–NO pathway may be essential to maintain the health of these animals.

The benefits of nitrate supplementation in the diets of swine are beginning to emerge (van den Bosch et al., 2019a; b; Doepker et al., 2021; van de Ligt et al., 2021). Van den Bosch et al. (2019a,b) studied the effects of dietary nitrate supplementation on reproductive outcomes in sows and their offspring when administered from day 108 of gestation and through day 5 of lactation. The study demonstrated that low-dose nitrate supplementation of 900 to 1,200 mg/kg nitrate in sows lowered the risk of piglet loss during farrowing. However, limited research exploring supplemental dietary nitrate has been conducted in growing pigs of any weight class. Further, no evaluation has been published regarding nitrate and nitrite levels in tissues in response to supplemental dietary nitrate and the potential impact of those levels on the safe consumption of those tissues by humans and animals.

Animal performance data in this study demonstrated that supplemental dietary nitrate did not adversely affect animal growth, feed intake, or overall health at dietary concentrations up to 2,000 mg/kg supplemental nitrate. These results are in alignment with the most current review by Doepker et al. (2021) who evaluated four studies with relatively high exposure values (>1,000 mg/kg body weight/day). Jahreis et al. (1986) determined a lowest-observed-adverse-effect level (**LOAEL**) of 1,400 mg/kg body weight/day using decreased weight gain and feed intake as the primary safety criteria. Koch et al. (1963) evaluated possible interactions between sodium nitrate and vitamin A intake which indicated a LOAEL of 1,300 mg/kg body weight/day but showed no effects on weight or feed efficiency. Tollett et al. (1960) also found no change in weight gain in swine fed dietary nitrate at levels equal to or less than 1,100 mg/kg body weight/day, and the study by Hutagalung et al. (1968) found no effects of nitrate administration on weight gain at 870 mg/kg body weight/day. For comparison, swine in this study were exposed to ~48 mg/kg body weight/day nitrate for the 2,000 mg/kg supplemental dietary nitrate treatment using an ADFI of ~3.2 kg and a mean body weight of ~131 kg. This level of dietary nitrate intake is well below the benchmark value of 410 mg/kg body weight/day established by EFSA (2020) and the greater level of 600 to 800 mg/kg body weight/day reported by Doepker et al. (2021).

Doepker et al. (2021) compared the points of departure across all relevant dietary nitrate studies and indicated that the most common safety endpoint investigated in swine was blood methemoglobin. Microbiota in the oral cavity and gastrointestinal tract convert nitrate to nitrite, which when absorbed, can oxidize the ferrous iron in hemoglobin, forming methemoglobin. The heme in methemoglobin is unable to bind oxygen, compromising the oxygen-carrying capacity of red blood cells and reducing blood oxygenation, potentially leading to hypoxia. In humans, 10% methemoglobin as a percentage of total hemoglobin is considered the threshold for nitrate toxicity, called methemoglobinemia (Wright et al., 1999). Thompson (2022) indicates that early signs of nitrate toxicosis in animals appear at 30% to 40% methemoglobin. In the current study, dietary nitrate supplementation did not influence hemoglobin or methemoglobin concentration. While the control group in this study had mean methemoglobin above the 10% threshold relevant to humans, no further treatment-related methemoglobin differences were observed between any of the treatment groups up to 2,000 mg/kg dietary nitrate. The methemoglobin levels were similar to those for unaffected pigs in a case study evaluating the interaction of potential nitrate toxicosis and classical swine fever where the authors reported a mean methemoglobin level of 15% (range 12% to 18%) for unaffected pigs (Sidhu et al., 2014) which indicates the 10% level may be a conservative threshold for nitrate toxicity in swine. The lack of nitrate toxicity was further supported by the observations of the overall health of the animals during the study (i.e., no signs of nitrate toxicity were observed), serum concentrations of nitrate and nitrite, and growth performance during twice daily physical observations. Therefore, the concentrations of methemoglobin recorded in this study were not indicative of nitrate toxicity. In addition, van de Ligt et al. (2021) concluded that levels of up to 6,000 mg/kg nitrate supplementation during the periparturient period did not increase the likelihood of methemoglobinemia occurrence, nor did it have any reported negative effects on the sows or piglets.

Nitrate and nitrite concentrations in liver and loin samples were below the detection limit for all treatments, which was 5 mg/kg for nitrite (as sodium nitrite) and 20 mg/kg for nitrate/nitrite N. These detection limit values are well below the limits for nitrate and nitrite in meat when using sodium nitrate as a feed preservative which are 364 mg/kg (calculated as 73% nitrate for 500 mg/kg sodium nitrate) and 133 mg/kg (calculated as 67% nitrate for 200 mg/kg sodium nitrite), respectively (21 CFR 172.170, FDA (U.S. Food and Drug Administration), 2023).

Serum nitrate and nitrite were also measured and found to be below the detection limit for all treatments, except for 3 pigs that were fed the greatest dietary nitrate concentration of 2,000 mg/kg supplemental dietary nitrate. These serum samples contained between 1 mg/kg (level of detection) and 10 mg/kg (level of quantitation) nitrate, which is within the normal range for diagnostic specimens (<10 mg/kg serum nitrate) and below the serum level for toxicity of 20 mg/kg for most animals (Thompson, 2022).

Previous research (Ascensao et al., 2018) demonstrated that supplemental dietary nitrate affects the color of the meat. In addition, nitrates have been used to cure meat for decades, whereby nitrates are reduced to nitrites, which react with myoglobin to produce nitrosomyoglobin, which is red in color (Froning et al., 1969). Myoglobin content also has been shown to influence the color of pork (Karamucki et al., 2013). Human consumption of nitrate (~808 mg, about 10 mg nitrate/kg body weight, as a single dose prior to a resistance exercise protocol) has been shown to alter oxygen utilization by the muscle (Garnacho-Castano et al., 2022) and to decrease basal metabolic rate after 3 daily consumptions of 0.1 mmol sodium nitrate per kg body weight (equivalent to 8.5 sodium nitrate/kg body weight and 6.2 mg nitrate/kg body weight; Larsen et al., 2014). Combined, these data suggest supplemental dietary nitrate may affect levels of myoglobin and its redox states. In this study, increasing supplemental dietary nitrate linearly increased total myoglobin in the loin, which was primarily driven by the increased myoglobin level in the loins of animals fed 2,000 mg/kg supplemental dietary nitrate. However, the redox forms of myoglobin, and specifically the metmyoglobin, did not differ across dietary nitrate levels, even at the greatest fed dose of 2,000 mg/kg supplemental dietary nitrate. Additional feeding studies should include instrumental color measurement (CIE color system) to measure the color of the meat in support of the beneficial effect of increased myoglobin on meat coloration.

## Conclusions

Collectively, the results from this study indicate that supplemental dietary nitrate up to 2,000 mg/kg is safe for consumption by finishing pigs immediately prior to slaughter and has no impact on the safety of consumption of the resultant meat products by humans and animals. Additionally, data obtained from the study provided new insights on the impact of supplemental dietary nitrate on myoglobin, which supports previous observations of meat with increased red color.

## References

[CIT0001] Amdahl, M. B., A. W. DeMartino, and M. T. Gladwin. 2020. Inorganic nitrite bioactivation and role in physiological signaling and therapeutics. Biol. Chem. 401:201–211. doi:10.1515/hsz-2019-0349 31747370

[CIT0002] American Meat Science Association. Meat Color Measurement Guidelines. Revised Dec 2012. www.meatscience.org

[CIT0003] AOAC Int. 2007. Official Methods of Analysis. 18th ed. Gaithersburg, MD: Assoc. Off. Anal. Chem. Int.

[CIT0004] Ascensao, A., B. Humphrey, and A. Van Wesel. 2018. Method for improving meat quality. International patent application number PCT/US2018/038931. Geneva, Switzerland: World Intellectual Property Organization, International Bureau

[CIT0005] Avery, A. A. 1999. Infantile methemoglobinemia: reexamining the role of drinking water nitrates. Environ. Health Perspect. 107:583–586. doi:10.1289/ehp.99107583 10379005 PMC1566680

[CIT0006] Bosch, H. M., A. B. Rosenfield, R. Huston, H. R. Shipman, and F. L. Woodward. 1950. Methemoglobinemia and Minnesota well supplies. J. Am. Water Works Assoc. 42:161–170. doi:10.1002/j.1551-8833.1950.tb18823.x.

[CIT0007] Bruning-Fann, C. S., and J. B. Kaneene. 1993. The effects of nitrate, nitrite, and N-nitroso compounds on animal health. Vet. Hum. Toxicol. 35:237–253. 8351799

[CIT0008] Bryan, N. S. and J. Loscalzo. 2017. Nitrite and nitrate in human health and disease. Nutrition and health series. Totowa, NJ, USA: Humana Press. doi:10.1007/978-3-319-46189-2

[CIT0009] Comly, H. H. 1945. Cyanosis in infants caused by nitrates in well water. J. Am. Med. Assoc. 129:112–116. doi:10.1001/jama.1945.02860360014004 3553637

[CIT0010] Doepker, C. L., M. M. Heintz, J. van de Ligt, and D. S. Wikoff. 2021. Review of potential risks associated with supplemental dietary exposure to nitrate-containing compounds in swine—a paradox in light of emerging benefits. Trans. Anim. Sci. 5:txab203. doi:10.1093/tas/txab203 PMC8665216 34909600

[CIT0011] EFSA CONTAM (European Food Safety Authority Panel on Contaminants in the Food Chain). 2020. Risk assessment of nitrate and nitrite in feed Amended 16 Nov 2020. EFSA J. 18:e06290. doi:10.2903/j.efsa.2020.6290 33173543 PMC7610142

[CIT0012] Emerick, R. J. 1974. Consequences of high nitrate levels in feed and water supplies. Fed. Proc. 33:1183–1187. 4599008

[CIT0013] FDA (U.S. Food and Drug Administration). Code of Federal Regulations. Title 21 Chapter I Subchapter B Part 172 (current as of June 6, 2023), https://www.ecfr.gov/current/title-21/section-172.170

[CIT0014] Froning, G. W., J. Daddario, T. Hartung, T. W. Sullivan, and R. M. Hill. 1969. Color of poultry meat as influenced by dietary nitrates and nitrites. Poult. Sci. 48:668–674. doi:10.3382/ps.0480668 5389882

[CIT0015] Garnacho-Castano, M. V., S. Sanchez-Nuno, L. Molina-Raya, T. Carbonell, J. L. Mate-Munoz, E. Pleguezuelos-Cobo, and N. Serra-Paya. 2022. Circulating nitrate-nitrite reduces oxygen uptake for improving resistance exercise performance after rest time in well-trained CrossFit athletes. Sci. Rep. 12:9761. doi:10.1038/s41598-022-13786-x 35690665 PMC9188609

[CIT0016] Ghasemi, A. 2022. Quantitative aspects of nitric oxide production from nitrate and nitrite. Excli J 21:470–486. doi:10.17179/excli2022-4727 35391922 PMC8983853

[CIT0017] Gilchrist, M., P. G. Winyard, and N. Benjamin. 2010. Dietary nitrate—good or bad? Nitric Oxide 22:104–109. doi:10.1016/j.niox.2009.10.005 19874908

[CIT0018] Gladwin, M. T., and D. B. Kim-Shapiro. 2008. The functional nitrite reductase activity of the heme-globins. Blood 112:2636–2647. doi:10.1182/blood-2008-01-115261 18596228 PMC2556603

[CIT0019] FASS. 2020. *Guide for the Care and Use of Agricultural Animals in Teaching and Research*. 4th ed. Federation of Animal Science Societies, Champaign, IL. https://www.asas.org/docs/default-source/default-document-library/agguide_4th.pdf?sfvrsn=56b44ed1_2

[CIT0020] Hord, N. G., Y. Tang, and N. S. Bryan. 2009. Food sources of nitrates and nitrites: the physiologic context for potential health benefits. Am. J. Clin. Nutr. 90:1–10. doi:10.3945/ajcn.2008.27131 19439460

[CIT0021] Hutagalung, R. I., C. H. Chaney, R. D. Wood, and D. G. Waddill. 1968. Effects of nitrates and nitrites in feed on the utilization of carotene in swine. J. Anim. Sci. 27:79–82. doi:10.2527/jas1968.27179x 5637670

[CIT0022] Jahreis, G., V. Hesse, F. Schöne, A. Hennig, and K. Gruhn. 1986. Effect of chronic dietary nitrate and different iodine supply on porcine thyroid function, somatomedin-C-level and growth. Exp. Clin. Endocrinol. 88:242–248. doi:10.1055/s-0029-1210603 3556413

[CIT0023] Karamucki, T., M. Jakubowski, A. Rybarczyk, and J. Gardzielewska. 2013. The influence of myoglobin on the colour of minced pork loin. Meat Sci. 94:234–238. doi:10.1016/j.meatsci.2013.01.014 23501256

[CIT0024] Koch, B. A., D. B. Parrish, and S. Sukhonthasarnpa. 1963. Effects of dietary NO2 and NO3 on growing swine. J. Anim. Sci. 22:840. (Abstr). http://hdl.handle.net/2097/8827.

[CIT0025] Larsen, F. J., T. A. Schiffer, B. Ekblom, M. P. Mattsson, A. Checa, C. E. Wheelock, T. Nyström, J. O. Lundberg, and E. Weitzberg. 2014. Dietary nitrate reduces resting metabolic rate: a randomized, crossover study in humans. Am. J. Clin. Nutr. 99:843–850. doi:10.3945/ajcn.113.079491 24500154

[CIT0026] Liu, Q., Y. Long, Y. F. Zhang, Z. Y. Zhang, B. Yang, C. Y. Chen, L. S. Huang, and Y. Su. 2021. Phenotypic and genetic correlations of pork myoglobin content with meat colour and other traits in an eight breed-crossed heterogeneous population. Animal. 15:100364. doi:10.1016/j.animal.2021.100364 34601209

[CIT0027] Lundberg, J., E. Weitzberg, and M. Gladwin. 2008. The nitrate–nitrite–nitric oxide pathway in physiology and therapeutics. Nat. Rev. Drug Discov. 7:156–167. doi:10.1038/nrd2466 18167491

[CIT0028] Lundberg, J. O., and M. Govoni. 2004. Inorganic nitrate is a possible source for systematic generation of nitric oxide. Free Radic. Biol. Med. 37:395–400. doi:10.1016/j.freeradbiomed.2004.04.027 15223073

[CIT0029] NRC. 2012. Nutrient requirements of swine. 11th rev. ed. Washington, DC: Natl. Acad. Press.

[CIT0030] Nyachoti, M. and E. Kiarie. 2010. Water in swine production: a review of its significance and conservation strategies. In: *Proceedings of the 24th* Manitoba Swine Seminar, pp. 217–232. https://www.thepigsite.com/articles/water-in-swine-production-a-review-of-its-significance-and-conservation-strategies

[CIT0031] Pawlak-Chaouch, M., J. Boissiere, F. X. Gamelin, G. Cuvelier, S. Berthoin, and J. Aucouturier. 2016. Effect of dietary nitrate supplementation on metabolic rate during rest and exercise in human: a systematic review and a meta-analysis. Nitric Oxide 53:65–76. doi:10.1016/j.niox.2016.01.001 26772523

[CIT0032] Powlson,D.S., T. M. Addiscott, N. Benjamin, K. G. Cassman, T.M. de Kok, H.L. van Grinsven, J. L. L’Hirondel, A. A. Avery, C. van Kessel. 2008. When does nitrate become a risk for humans? J. Environ. Qual. 37:291–295. doi:10.2134/jeq2007.0177 18268290

[CIT0033] Sidhu P. K. , V. Mahajan, S. Verma, Ashuma, M. P. Gupta. 2014. Toxicological and pathological review of concurrent occurrence of nitrite toxicity and Swine Fever in pigs. Toxicol. Int. 21:186–190. doi:10.4103/0971-6580.139806 25253929 PMC4170561

[CIT0034] Smith, H. C., V. E. Lovell, R. Reppert, and D. Griswold. 1959. Nitrate poisoning in swine. Vet. Med. 54:547–550.

[CIT0035] Standard Methods Committee of the American Public Health Association, American Water Works Association, and Water Environment Federation. 4500-no3− nitrogen (nitrate). In: Lipps W. C., T. E. Baxter, and E. Braun-Howland, editors. Standard methods for the examination of water and wastewater. Washington DC: APHA Press. doi:10.2105/SMWW.2882.089

[CIT0036] Tang, J., C. Faustman, and T. A. Hoagland. 2004. Krzywicki revisited: equations for spectrophotometric determination of myoglobin redox forms in aqueous meat extracts. J. Food Sci. 69:717–720. doi:10.1111/j.1365-2621.2004.tb09922.x

[CIT0037] Thompson, L. J. 2022. Nitrate and nitrate poisoning in animals. In: Merck veterinary manual. Kenilworth (NJ): Merck & Co., Inc. https://www.merckvetmanual.com/toxicology/nitrate-and-nitrite-poisoning/nitrate-and-nitrite-poisoning-in-animals

[CIT0038] Tollett, J. T., D. E. Becker, A. H. Jensen, and S. W. Terrill. 1960. Effect of dietary nitrate on growth and reproductive performance of swine. J. Anim. Sci. 19:1297. doi:10.2527/jas1960.1941216x

[CIT0039] USEPA. 1991. Nitrate (CASRN14797-55-8). IRIS. https://iris.epa.gov/static/pdfs/0076_summary.pdf

[CIT0040] van de Ligt, J., K. Saddoris-Clemons, S. Norton, M. Davis, and C. Doepker. 2021. Impact of calcium nitrate supplementation on the oxygen-carrying capacity of lactating sows and their offspring. Trans. Anim. Sci. 5:1–11. doi:10.1093/tas/txab217 PMC8714184 34988377

[CIT0041] van den Bosch, M., J. Wijnen, I. B. van de Linde, A. A. M. van Wesel, D. Melchior, B. Kemp, H. van den Brand, and C. Clouard. 2019b. Effects of maternal dietary nitrate supplementation on farrowing and placental characteristics, level of asphyxiation at birth and piglet vitality. Theriogenology. 129:1–7. doi:10.1016/j.theriogenology.2019.01.033 30784789

[CIT0042] van den Bosch, M., J. Wijnen, I. B. van de Linde, A. A. M. van Wesel, D. Melchior, B. Kemp, C. Clouard, and H. van den Brand. 2019a. Effects of maternal dietary nitrate supplementation during the perinatal period on piglet survival, body weight, and litter uniformity. Trans. Anim. Sci. 3:464–472. doi:10.1093/tas/txy137 PMC7200544 32704817

[CIT0043] van’t Hof, R. J., and S. H. Ralston. 2001. Nitric oxide and bone. Immunology. 103:255–261. doi:10.1046/j.1365-2567.2001.01261.x 11454054 PMC1783253

[CIT0044] Walton, G. 1951. Survey of literature relating to infant methemoglobinemia due to nitrate-contaminated water. Am. J. Public Health Nations Health. 41:986–996. doi:10.2105/ajph.41.8_pt_1.986 14847023 PMC1525621

[CIT0045] Weitzberg, E., M. Hezel, and J. O. Lundberg. 2010. Nitrate-nitrite-nitric oxide pathway: implications for anesthesiology and intensive care. Anesthesiology. 113:1460–1475. doi:10.1097/ALN.0b013e3181fcf3cc 21045638

[CIT0046] Wikoff, D. S., C. Thompson, J. Rager, G. Chappell, S. Fitch, and C. Doepker. 2018. Benefit-risk analysis for foods (BRAFO): evaluation of exposure to dietary nitrates. Food Chem. Toxicol. 120:709–723. doi:10.1016/j.fct.2018.08.031 30134152

[CIT0047] Wimalawansa, S. J. 2020. Targeting nitric oxide for bone disease. In: Zaidi M. editor. Encyclopedia of Bone Biology. Cambridge (MA): Academic Press; p. 666–696.

[CIT0048] Wright, R. O., W. J. Lewander, and A. D. Woolf. 1999. Methemoglobinemia: etiology, pharmacology, and clinical management. Ann. Emerg. Med. 34:646–656. doi:10.1016/s0196-0644(99)70167-8 10533013

[CIT0049] Wu, G., W. B. Fuller, G. A. Johnson, and Y. Hou. 2018. Board-invited review: arginine nutrition and metabolism in growing, gestating, and lactating swine. J. Anim. Sci. 96:5035–5051. doi:10.1093/jas/sky377 30445424 PMC6276552

